# The Pessimist Again

**Published:** 1920-03-06

**Authors:** 


### THE PESSIMIST AGAIN.

When a paragraph in a lay paper upon some general question reads like panic, the practised observer infers that the public is being "prepared" for some official move, or that the journal which publishes the paragraph is about to proclaim some new policy. We have observed before that the "medical correspondent" of *The Times* is not always remarkable for sober judgment, and were therefore hardly startled to read on February 27 a paragraph headed "Hospital Crisis: Need for Government Action." After alluding to present difficulties, while suppressing any reference to the successful efforts now being made throughout the country on voluntary lines to overcome them, the "medical correspondent" says: "It is quite evident that the Government must frame a large policy and prepare to carry it out." He is good enough to add that "while no desire exists to supplant the voluntary system, this system is inadequate to present needs." Therefore, he hastens to repeat, "the time has arrived when a measure embodying the present necessities must be introduced into Parliament." If this is not damning the voluntary system with the faintest of praise, it is meaningless. In reality of course it is either a kite flown to test the chances of some already determined measure, or a decoy to prepare the public for a fresh advocacy of State control.

In the present mood of the nation such kites should not be ignored. The mood in favour of bureaucracy, which is one of the symptoms of weariness resulting from the war, is in the ascendant still, and the periodic outcries against this or that example of departmental waste or failure usually end in a demand for the creation of another department to sweep up the dust created by the first. The bureaucracy is dead, long live the bureaucracy! In this mood ignorance flourishes; and the above remarks display a significant ignorance of the facts. To say that "the voluntary system is inadequate to the present needs" is simply untrue. What is true is merely that the voluntary system, *as it existed before the war*, is inadequate to the needs which the war has left behind it.

But the voluntary system, because of the war, has undergone a change; and the war, which created new demands upon it, created also a response to meet them. That response is not complete: how could it be at present? But its influence is being felt every day in every corner of the country. During the past few months, especially during the latest period, The Hospital has been filled with news of the launching of successful schemes to expand the resources of the voluntary hospitals. The grant of the surplus funds of the Red Cross Society to the voluntary hospitals was no isolated phenomenon. The Society and its numerous branches are pulsing with the effort to direct their war-time organisations towards the maintenance of the voluntary hospitals. The alliance formed during the war has been cemented, and those fully apprised of recent plans know that it will be extended and enduring. Plans are already afoot, and in active being, whereby the Red Cross organisation throughout the country shall become a new and permanent buttress for the voluntary hospitals. The latter on their own initiative have already created large funds in the chief centres of the provinces. We have recorded the raising of £100,000 in the space almost of a few days at Cardiff, of the great scheme for future maintenance successfully created at Leeds, of definite progress towards the co-ordination of the hospital service in the Birmingham area; and of many more schemes less completely advanced. Behind all these stand the Red Cross and Sir Arthur Stanley in the background, from whom more will be heard before the end of the present year. The facts which we mention here are significant not only in themselves but for their tendency, which is seen in the voluntary system generally to be a new impulse of growth. The *élan* of that movement is unmistakable. Its achievements will be measurable before long; and those who are unconscious of it, or who affect to ignore its importance, simply confess themselves to be disqualified for the serious discussion of hospital affairs.

